# Differential Responses of a Coastal Prokaryotic Community to Phytoplanktonic Organic Matter Derived from Cellular Components and Exudates

**DOI:** 10.1264/jsme2.ME20033

**Published:** 2020-06-17

**Authors:** Hiroaki Takebe, Kento Tominaga, Kentaro Fujiwara, Keigo Yamamoto, Takashi Yoshida

**Affiliations:** 1 Graduate School of Agriculture, Kyoto University, Kitashirakawa-Oiwake, Sakyo-ku, Kyoto, 606–8502, Japan; 2 Research Institute of Environment, Agriculture and Fisheries, Osaka Prefecture, 442, Shakudo Habikino, Osaka, 583–0862, Japan

**Keywords:** marine prokaryotes, phytoplankton bloom, microcosm, 16S rRNA gene, metagenomic data

## Abstract

The phytoplanktonic production and prokaryotic consumption of organic matter significantly contribute to marine carbon cycling. Organic matter released from phytoplankton via three processes (exudation of living cells, cell disruption through grazing, and viral lysis) shows distinct chemical properties. We herein investigated the effects of phytoplanktonic whole-cell fractions (WF) (representing cell disruption by grazing) and extracellular fractions (EF) (representing exudates) prepared from *Heterosigma akashiwo*, a bloom-forming *Raphidophyceae*, on prokaryotic communities using culture-based experiments. We analyzed prokaryotic community changes for two weeks. The shift in cell abundance by both treatments showed similar dynamics, reaching the first peak (~4.1×10^6^‍ ‍cells‍ ‍mL^–1^) on day 3 and second peak (~1.1×10^6^‍ ‍cells‍ ‍mL^–1^) on day 13. We classified the sequences obtained into operational taxonomic units (OTUs). A Bray-Curtis dissimilarity analysis revealed that the OTU-level community structure changed distinctively with the two treatments. Ten and 13 OTUs were specifically abundant in the WF and EF treatments, respectively. These OTUs were assigned as heterotrophic bacteria mainly belonging to the *Alteromonadales* (*Gammaproteobacteria*) and *Bacteroidetes* clades and showed successive dynamics following the addition of organic matter. We also analyzed the dynamics of these OTUs in the ocean using publicly available metagenomic data from a natural coastal bloom in Monterey Bay, USA. At least two WF treatment OTUs showed co-occurrence with *H. akashiwo*, indicating that the blooms of *H. akashiwo* also affect these OTUs in the ocean. The present results strongly suggest that the thriving and dead cells of uninfected phytoplankton differentially influence the marine prokaryotic community.

Marine phytoplankton are estimated to be responsible for approximately 50% of global carbon fixation (Field *et al.*, 1998), and a large proportion of marine primary products (15–80%) is consumed by heterotrophic microbes (Azam *et al.*, 1983; Worden *et al.*, 2015). Heterotrophic bacteria also contribute to the remineralization of organic matter, which is taken up by phytoplankton (Buchan *et al.*, 2014). Therefore, the relationship between phytoplankton and heterotrophic communities needs to be examined in more detail to obtain a better understanding of the marine biogeochemical cycle.

Recent studies revealed the daily succession of abundant heterotrophic prokaryotes accompanied by distinct gene function repertoires (*e.g.* carbohydrate and amino acid metabolism-related genes) during and after the natural phytoplankton bloom, suggesting that changes in the availability of organic matter shape the prokaryotic community (Teeling *et al.*, 2012; Wemheuer *et al.*, 2015; Needham and Fuhrman, 2016; Wöhlbrand *et al.*, 2017). Thus, the characterization of phytoplankton-derived organic matter is required for understanding the relationship between phytoplankton and heterotrophic communities (Buchan *et al.*, 2014).

The chemical properties of phytoplankton-derived organic matter (such as amino acids, monosaccharides, and organic sulfur compounds) differ among lineages (Brown, 1991), and even among growth phases within single species (Tada *et al.*, 2017). Previous culture-dependent studies demonstrated that these chemical differences are potential factors that shape the bacterial community (Sarmento *et al.*, 2013; Landa *et al.*, 2014; Tada *et al.*, 2017). However, limited information is currently available on the effects of differences in the release processes of organic matter from phytoplanktonic cells on the shape of the prokaryotic community.

Phytoplankton cells generally release organic matter via exudation (Fogg, 1983), disruption caused by predation (Strom *et al.*, 1997), or viral lysis (Suttle, 2005). Exudates released from healthy living cells contain photosynthetic products that exceed the requirements for cell activity (Fogg, 1983) and signaling compounds (Pohnert, 2010), including defensive compounds used against predators (Ianora *et al.*, 2004) and allelopathic compounds against competitors (Prince *et al.*, 2008). Phytoplanktonic release via exudation and the bacterial assimilation of organic matter account for up to 40% of total marine primary production (Fogg, 1983). Sloppy feeding by grazers causes the death and disruption of phytoplanktonic cells and contributes to the formation of organic matter reservoirs (Strom *et al.*, 1997). This leakage of organic matter may supply some parts of organelles or membranes in the ocean at the soluble level (Ma *et al.*, 2018). A co-culture experiment using phytoplankton, grazers, and bacteria showed that grazing increased the concentration of dissolved organic matter (Strom *et al.*, 1997). Viral infection is another mechanism that is responsible for the release of organic matter with distinct chemical properties (Rosenwasser *et al.*, 2016). Viral lysis may affect the mortality of marine prokaryotes and eukaryotes (Suttle, 2005), resulting in the release of organic matter from host cells (Yoshida *et al.*, 2018) and the supply of substrates for heterotrophic prokaryotes (Zimmerman *et al.*, 2020). Viral infections often redirect host metabolic pathways during infection using auxiliary metabolic genes to facilitate their replication (Rosenwasser *et al.*, 2014). A recent culture-based study revealed that the composition of the chemical compounds (proteins, carbohydrates, and lipids) of organic matter released via these three processes differed, even among cells of a single strain (Ma *et al.*, 2018). Therefore, a detailed understanding of the effects of chemically distinct organic matter on prokaryotic communities will provide important insights into the mutual, but complex, relationships between the state of phytoplanktonic cells (life or death) and prokaryotes.

We herein focused on organic matter released from healthy phytoplankton cells, and fractionated organic matter from a single strain of uninfected phytoplankton into extracellular fractions (EF) and whole-cell fractions (WF), representing exudates of living cells and cell disruption by grazing, respectively. We then investigated the effects of WF and EF prepared from *Heterosigma akashiwo*, a global bloom-forming species belonging to *Raphidophyceae* (Chang *et al.*, 1990; Nagasaki *et al.*, 1994a, Needham and Fuhrman, 2016), on coastal prokaryotic communities using culture-based experiments. We analyzed rapid changes in the prokaryotic community following the addition of WF or EF using 16S rRNA targeting analyses and followed up on the results of our culture experiments using environmental genomic data from a natural coastal phytoplankton bloom in Monterey Bay, USA (Nowinski *et al.*, 2019) to establish whether WF- and/or EF-responding species have a relationship with the natural bloom of *H. akashiwo*.

## Materials and Methods

### Culturing of phytoplankton and preparation of organic matter

*H. akashiwo* strain NIES-293 was obtained from the National Institute for Environmental Studies (NIES, Tsukuba, Japan) and grown axenically in 300‍ ‍mL of f/2 medium (Guillard and Ryther, 1962) at 20°C under a 10/14-h light/dark photocycle at 40 μmol photon m^–2^ s^–1^. Previous studies on natural blooms of *H. akashiwo* evaluated its abundance based on cell counts, which ranged between 10^4^ and 10^5^‍ ‍cells‍ ‍mL^–1^, but not carbon concentrations (Nagasaki *et al.*, 1994a, 1994b; Kim *et al.*, 1998; Tomaru *et al.*, 2004). Therefore, we examined the amount of WF or EF needed for the inoculum based on the cell abundance of *H. akashiwo* and considered 1.5×10^4^‍ ‍cells‍ ‍mL^–1^ to be reasonable to represent the bloom in our experiments based on previous findings (Nagasaki *et al.*, 1994a, 1994b; Kim *et al.*, 1998; Tomaru *et al.*, 2004). One‍ ‍hundred and fifty milliliters of the phytoplankton cell culture in the exponential phase (cultured for 14‍ ‍d and reaching 1.5×10^4^‍ ‍cells‍ ‍mL^–1^) was filtered through polycarbonate membrane filters (diameter of 47‍ ‍mm, pore size of 0.2 μm; Toyo Roshi Kaisha) to remove cells. The resulting filtrate was defined as the extracellular fraction (EF). The remaining culture (150‍ ‍mL) was centrifuged at 420×*g* for 10‍ ‍min (High Capacity Bench-top Centrifuge LC-220; TOMY SEIKO). The pellet was washed with f/2 medium twice and resuspended in 150‍ ‍mL of fresh f/2 medium. Washed cells were homogenized using an Ultrasonic Disruptor (UD-211; TOMY SEIKO) at 30 W for 10 cycles of 15‍ ‍s on ice. Cell breakage was confirmed by microscopic observations. Although some debris including membranes may have remained through the cell breakage process, these debris were not removed. The inclusion of this debris appears to be suitable for reconstructing environmental conditions; previous studies reported that grazing caused the release of particulate organic matter as well as dissolved organic matter, supplying substrates for particulate-associated prokaryotes (Lee and Fisher, 1994), which were included in the prokaryotic fraction defined in this study (0.2–3.0‍ ‍μm) (Hollibaugh *et al.*, 2000; Needham and Fuhrman, 2016; Orsi *et al.*, 2016). Thus, we used this lysate in subsequent culture experiments and defined it as the whole-cell fraction (WF).

### Experimental set-up

Approximately 5 L of seawater was collected from the surface (5 m) of Osaka Bay, Japan (N 34°19'28", E 135°7'15") in November 2017. It was pre-filtered through polycarbonate membrane filters (diameter of 142‍ ‍mm, pore size of 3.0 μm; Millipore) to remove eukaryotic cells. In culture-based experiments, 270‍ ‍mL of pre-filtered seawater was again filtered through polycarbonate membrane filters (diameter of 47‍ ‍mm, pore size of 0.2 μm; Millipore), and prokaryotic cells on the filters were suspended in 270‍ ‍mL of autoclaved aged seawater (seawater collected in Osaka Bay was left in a dark room at 4°C for more than 6 months and then filtered through a polycarbonate membrane filter [diameter of 142‍ ‍mm, pore size of 0.2 μm; Millipore]) in 500-mL flasks (washed with 6 N HCl and Milli-Q water before use to remove residual organic matter). The input cell abundance was considered to exceed 1.5×10^6^‍ ‍cells‍ ‍mL^–1^ based on a previous study that had estimated prokaryotic abundance in Osaka Bay (Naganuma and Miura, 1997). Thirty milliliters of WF or EF was added to seawater samples for the bacterial response analysis, and an equivalent volume of f/2 medium was added to another flask as a control. Each treatment was designed in triplicate (designated as replicates I-III in each treatment). These samples were incubated at 20°C for 14‍ ‍d under a 10/14-h light/dark photocycle at 40 μmol photon m^–2^ s^–1^ to reflect the natural environmental conditions of Osaka Bay in November (data from the National Astronomical Observatory of Japan; https://eco.mtk.nao.ac.jp/index.html.en).

### Subsampling

Incubation flasks were mixed once a day before subsampling. Samples were collected on days 1, 3, 5, 7, 9, 11, and 13 to capture the rapid transition of the prokaryotic community. Sixty-three samples were obtained and one (EF-treatment replicate III, day 3) was lost because of a technical error. In cell counting, 1,920‍ ‍μL of each sample was collected and flow cytometry was used to monitor cell numbers in the flasks. Cells preserved in glutaraldehyde (1% final concentration) were stained with SYBR^®^ Green I (final concentration 1×; Thermo Fisher Science). At least 10,000 cells (measurement time >15 s) were counted in each sample using the S3e Cell Sorter (Bio Rad). The total cell number was analyzed from these data using FlowJo (Becton, Dickinson and Company); Mann–Whitney U tests were performed with the Bonferroni correction for comparisons of cell abundance in the three treatments after the Shapiro–Wilk test to confirm the non-normal distribution of data in each treatment. In the prokaryotic community structure analysis, 2-‍mL samples were collected. Samples were filtered through polycarbonate membrane filters (diameter of 13‍ ‍mm, pore size of 0.2‍ ‍μm; Toyo Roshi Kaisha) and the filters were stored at –30°C until DNA extraction. To analyze the prokaryotic community composition in original seawater used as a prokaryotic inoculum, 15‍ ‍mL of original seawater was filtered through polycarbonate filters (diameter of 13‍ ‍mm, pore size of 0.2 μm; Toyo Roshi Kaisha), and the filters were stored at –30°C until DNA extraction.

### DNA extraction and sequencing

DNA was extracted using slightly modified versions of previously published lysis and purification methods (Frias-Lopez *et al.*, 2008). Briefly, filters were thawed, placed in microcentrifuge tubes, mixed with a 5‍ ‍mg mL^–1^ solution of lysozyme (Sigma-Aldrich) in 1‍ ‍mL of lysis buffer, and incubated at 37°C for 30‍ ‍min. Sodium dodecyl sulfate (final concentration of 1%) was then added to the tubes, followed by proteinase K (TaKaRa Bio) at a final concentration of 0.5‍ ‍mg mL^–1^. The filters were incubated at 55°C for 20‍ ‍min, followed by a further incubation at 70°C for 5‍ ‍min to induce cell lysis. The aqueous phase was transferred into new tubes. The lysate was removed from the aqueous phase using a combination of phenol:chloroform:isoamyl alcohol (25:24:1) and chloroform:isoamyl alcohol (24:1) protocols. The purified aqueous phase was washed with 70% ethanol and concentrated using sterile Milli-Q water to isolate DNA. In each sample, a 30-μL aliquot was obtained and stored at –30°C. To detect bacteria and archaea in samples, 16S rDNA was amplified using a primer set based on the V3–V4 hypervariable region of prokaryotic 16S rRNA genes (Takahashi *et al.*, 2014) with added overhang adapter sequences at each 5′-end, according to the 16S sample preparation guide (https://support.illumina.com/content/dam/illumina-support/documents/documentation/chemistry_documentation/16s/16s-metagenomic-library-prep-guide-15044223-b.pdf). Amplicons were sequenced using the MiSeq Reagent kit, version 3 (2×300-bp read length; Illumina).

### Sequence data processing and statistical analysis

Primer regions were removed using USEARCH (Edgar, 2010). The quality of reads was scanned using a 50-base-wide sliding window analysis, and reads were then trimmed when the average quality per base was smaller than 20 using Trimmomatic –0.35 (Bolger *et al.*, 2014) with the -phred33 quality scores option. The other parameters were set according to the original paper on this software (Bolger *et al.*, 2014). Trimmed complementary sequences were merged using the --fastq_mergepairs command in VSEARCH version 2.4.3 (Rognes *et al.*, 2016) and when the length of merged reads was smaller than 200 bases or the minimum quality per base was below 20, these reads were discarded. Chimeras were removed using the --uchime_ref command in VSEARCH. The remaining merged reads were clustering using VSEARCH to form operational taxonomic units (OTUs) with a sequence identity threshold of 99%. Singleton OTUs were discarded at this stage. Representative sequences of the remaining OTUs were aligned to the SILVA ribosomal RNA gene database (release 132) (Quast *et al.*, 2013) with SINA using the ARB software package (Pruesse *et al.*, 2012). Sequences were then assigned to taxonomic clades using the ARB export node display set-up (NDS) function. OTUs assigned for mitochondria (0.01% in each sample on average) or chloroplasts (20% in each sample on average) were removed from further analyses. To minimize differences in sequence depths, we restricted the range of the number of sequences from 10,000 to 199,999 reads (Needham and Fuhrman, 2016). Samples with less than 10,000 reads were removed (1 sample; EF-treatment replicate I, day 3), and samples with more than 199,999 reads were rarefied to 199,999 reads using the “vegan” package in R (2 samples; WF-treatment replicate II, day 3 and replicate III, day 13). The relative abundance of each OTU was calculated as the percentage of sequences that belonged to that OTU among all sequences in a sample. To detect abundant species among these OTUs, we adopted the criteria that OTUs with relative abundance exceeding 0.4% on average or 2.5% on at least 1‍ ‍d of the culture were defined as “abundant OTUs” as described previously (Needham and Fuhrman, 2016). We confirmed that the total relative abundance of abundant OTUs defined with these criteria exceeded 69% in all samples. This result was sufficient to investigate abundant species in the flask and, thus, we applied these criteria in subsequent analyses.

Rarefaction curves were calculated for all samples using the “iNEXT” (Hsieh *et al.*, 2016) package in R (R Core Team, 2017). Before calculation, 50,000 reads were randomly extracted from each sample (samples with fewer than 50,000 reads were compensated using the “extrapolation” function in “iNEXT”). Alpha- and beta-diversity analyses were performed on 61 samples. To normalize the data size, 10,000 reads were randomly extracted from each sample. The Shannon index was calculated using the “vegan” (Oksanen *et al.*, 2013) package in R. Mann–Whitney U tests were performed with Bonferroni corrections for comparisons of the Shannon index in the three treatments after the Shapiro–Wilk test to confirm the non-normal distribution of data in each treatment. Bray-Curtis dissimilarities were generated using “vegan” in R for pairwise comparisons of the prokaryotic communities in all flasks, which were then visualized by a principal coordinate analysis (PCoA) using the “stats” package in R. An analysis of similarity (ANOSIM) was performed with the Bray-Curtis dissimilarities score to assess the significance of differences between early (day 1–3) and late (day 11–13) samples and that among late samples between the WF and EF treatments using the R package “vegan”.

### Investigation of interactions between *H. akashiwo* and abundant OTUs in the environment

To investigate whether the data from our experimental design was applicable to ecological interactions of *H. akashiwo* and abundant OTUs in the ocean, we examined the dynamics of *H. akashiwo* and abundant OTUs obtained in the present study during a natural phytoplankton bloom. To achieve this, we searched for publicly available environmental sequence data satisfying all of the following criteria: 1) blooms of *H. akashiwo* were detected, 2) sampling was conducted within a 2-day interval from pre- and post-blooms to maintain similar time scales with our culture-based experiment, and 3) the sequenced region was identical to that in this study, the 16S rRNA V3–V4 region, in order to map reads in environmental samples on our experimental dataset. A previous study on a coastal phytoplankton bloom at Monterey Bay (USA) (Nowinski *et al.*, 2019) provided the only dataset that met our requirements, and, thus, we used these sequence data in subsequent analyses. The 18S rRNA gene sequence of *H. akashiwo* NIES-293 (DQ470658.1) (Ki and Han, 2007) was downloaded from GenBank (Benson *et al.*, 2012). Raw reads of the 18S rRNA and 16S rRNA genes obtained from the phytoplankton bloom between 26 September and 16 November, 2016 were downloaded from the NCBI Sequence Read Archive (PRJNA533622) (Nowinski *et al.*, 2019). These reads were analyzed after the removal of chimeras with the pipeline described in the previous section. The remaining reads of the 18S rRNA and 16S rRNA genes were mapped to the sequence of *H. akashiwo* NIES-293 and the representative sequences of abundant OTUs with 99% identity using VSEARCH, respectively. We defined eukaryotic strains that shared more than 99% identity with the NIES-293 strain as *H. akashiwo* relatives. Replicates of samples from the same day were merged, and average abundance was used in analyses.

### Phylogenetic analyses based on the 16S rRNA gene

We retrieved the 16S rRNA gene sequences of *Gammaproteobacteria* from the SILVA ribosomal RNA gene database (release 132) to construct a reference phylogenetic tree. Sequences were aligned using MAFFT/7.427 (Katoh and Standley, 2013) by the L-INS-i method (--localpair algorithm combined with 1,000 cycles of iterative refinement), and gap positions were removed automatically using trimAl/1.4.1 (Capella-Gutierrez *et al.*, 2009) with the -gappyout option. The other parameters were default settings. Phylogenetic reconstructions were performed using the approximately-maximum-likelihood method using FastTree/2.1.11 (Price *et al.*, 2010) and visualized with iTOL version 4.4.2 (Letunic and Bork, 2019). The robustness of the topology of the phylogenetic trees was evaluated by a bootstrap analysis from 100 runs. Abundant OTUs assigned as *Alteromonadales* were classified using pplacer/1.1.alpha19 (Matsen *et al.*, 2010).

### Nucleotide sequence accession numbers

The nucleotide sequence data obtained from the 16S rRNA gene targeting analysis in the present study have been submitted to the DNA Data Bank of Japan (DDBJ) under accession number DRA009375.

## Results

### Shift in abundance of prokaryotic communities in response to organic matter

**Fig. 1.**
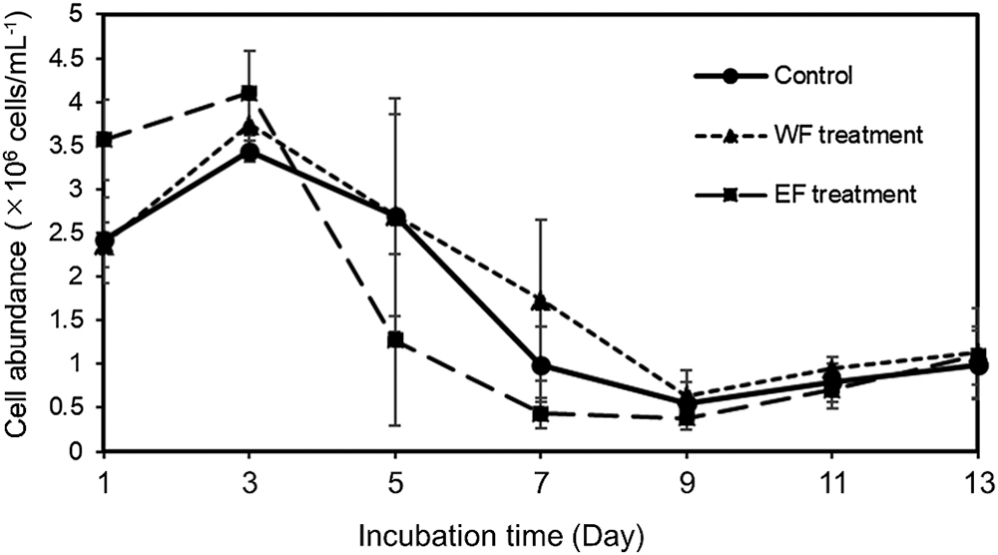
Cell abundance shifts in the prokaryotic community during the culture experiment. Cell counts were analyzed using flow cytometry. Average cell numbers in triplicate flasks are shown. Error bars indicate standard errors (regarding the sample on day 3 in the EF treatment, *n*=2).

We investigated shifts in prokaryotic cell abundance in response to WF and EF from *H. akashiwo*. Cell density in the control and WF- or EF-treated cultures showed similar dynamics (Fig. 1). Average cell abundance calculated from data in samples on day 1–13, among the three treatments was not significantly different (the Mann–Whitney U test, *P*>0.05). Cell density in the control culture increased to the first maximum of 3.4 (±0.1)×10^6^‍ ‍cells‍ ‍mL^–1^ on day 3, decreased until day 9, and finally reached the second maximum of 1.0 (±0.4)×10^6^‍ ‍cells‍ ‍mL^–1^ on day 13. In WF and EF-treated cultures, cell density reached the first maximum of 3.7 (±0.3)×10^6^‍ ‍cells‍ ‍mL^–1^ (WF treatment) and 4.1 (±0.5)×10^6^‍ ‍cells‍ ‍mL^–1^ (EF treatment) on day 3, and then decreased before reaching the second peak of 1.1 (±0.5)×10^6^‍ ‍cells‍ ‍mL^–1^ (WF treatment) and 1.1 (±0.3)×10^6^‍ ‍cells‍ ‍mL^–1^ (EF treatment) on day 13. Based on these results, we divided the culture period into three phases: early (day 1 to 3; cell density increasing), middle (day 5 to 9; cell density decreasing), and late (day 11 to 13; cell density increasing). The reasons for the shift in prokaryotic abundance remained unclear. However, the shift in cell abundance indicated that prokaryotes increased in response to the addition of f/2 medium and/or organic matter by day 3–5, decreased possibly due to a lack of substrates by day 9, and then increased again, potentially by utilizing the organic matter produced by phototrophic prokaryotes or released from prokaryotic carcasses.

Density plot patterns analyzed by flow cytometry also revealed that the values of FSC (indicating the size of cells) and SSC (indicating the complexity of the cell structure) in dominant cells changed over the course of the experiment (Fig. S1). In control sample replicate I, two dense plot groups emerged in the early phase; these plots were dispersed in the middle phase, and plots indicating less complex and smaller cells were detected in the late phase. This pattern was common across treatments. Comparisons of cytograms on day 13 revealed that plot patterns in the EF treatment slightly differed from those in the other treatments (Fig. S1), even after culturing for the same period.

### Shifts in the prokaryotic community and diversity in response to organic matter

In the original seawater sample, 151,101 reads and 2,652 OTUs were obtained (Table S1). During cultivation, 78,910, 165,193, and 44,141 reads and 792, 1,043, and 588 OTUs were obtained per flask in the control, WF treatment, and EF treatment, respectively (Table S1). To test the sufficiency of sequence depths, we performed a rarefaction analysis, which revealed that the number of OTUs present in the control, WF treatment, and EF treatment slightly increased with sequencing depths (Fig. S2).

**Fig. 2.**
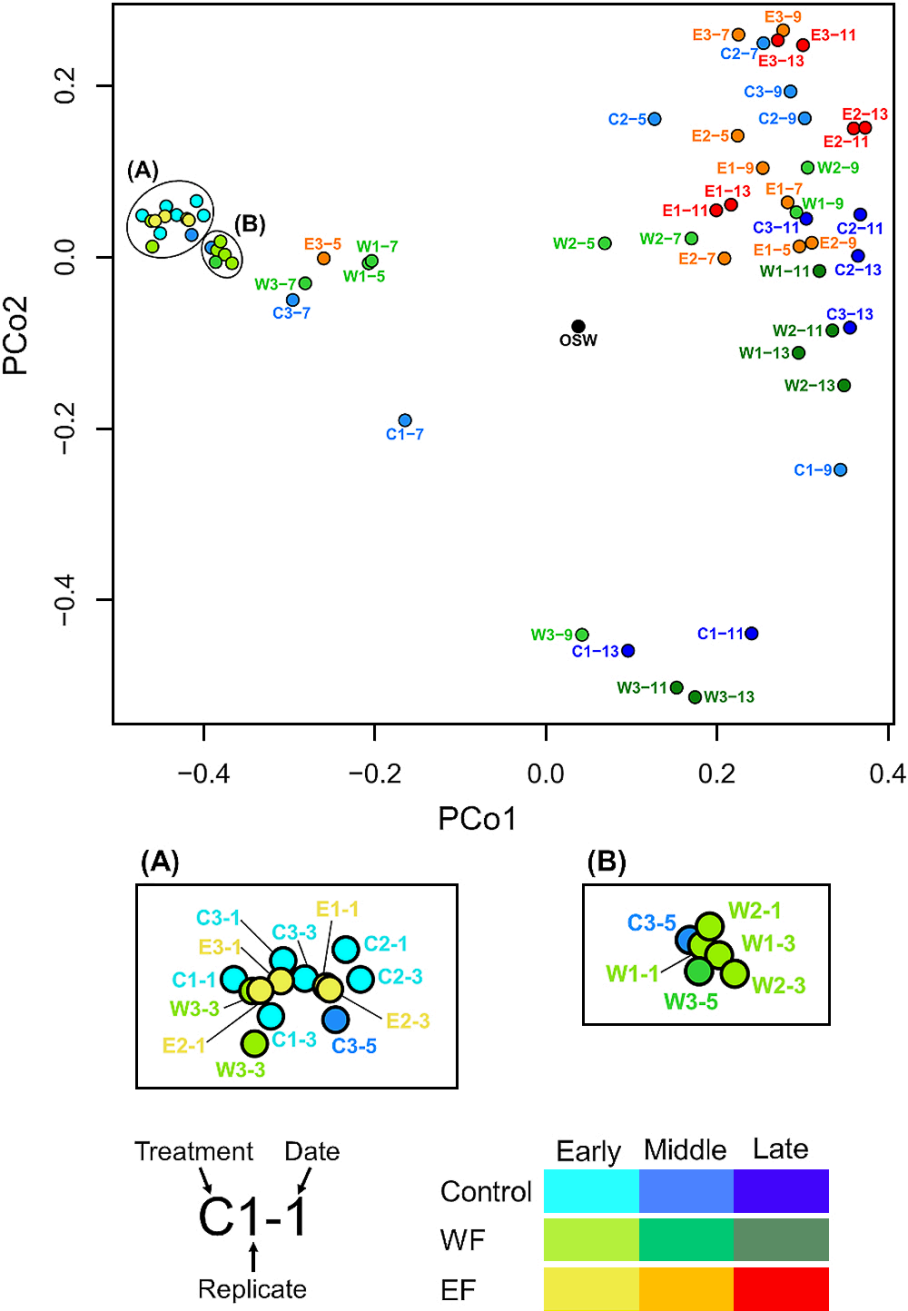
Comparisons of beta-diversity among three treatments. Bray-Curtis dissimilarity among all samples was calculated using “vegan” package in R, then illustrated by a Principal Coordinate Analysis (“stats” package in R). Sample IDs are distinguished by colors based on the treatments and culture periods. OSW: original seawater sample. Enlarged figures of areas (A) and (B) were shown separately.

Alpha- and beta-diversity analyses were performed based on equal numbers of sequences (10,000 reads). The Shannon index (representing alpha-diversity) of the original seawater sample was 4.90 and then shifted to 2.20–3.50 in all three treatments by day 1 (Fig. S3A). The average Shannon index (calculated from data in samples on day 1–13) of the three treatments was not significantly different (the Mann–Whitney U test, *P*>0.05) (Fig. S3B). Beta-diversity analyzed by Bray-Curtis dissimilarity revealed that prokaryotic communities in all three treatments markedly differed from that in the original seawater sample on day 1 (Fig. 2). Samples in the early and late phases were clearly separated in all treatments (ANOSIM, *P*<0.005). When we compared plot patterns among treatments, the plots of all three treatments were closer in the early phase and became distinctive after the middle phase. While the middle and late phase samples of the control treatment were spread out over a larger area, those of the WF and EF samples were closer together with the treatment, except for replicate III in the WF treatment. The late phases of the WF and EF samples were plotted separately (ANOSIM, *P*<0.005).

**Fig. 3.**
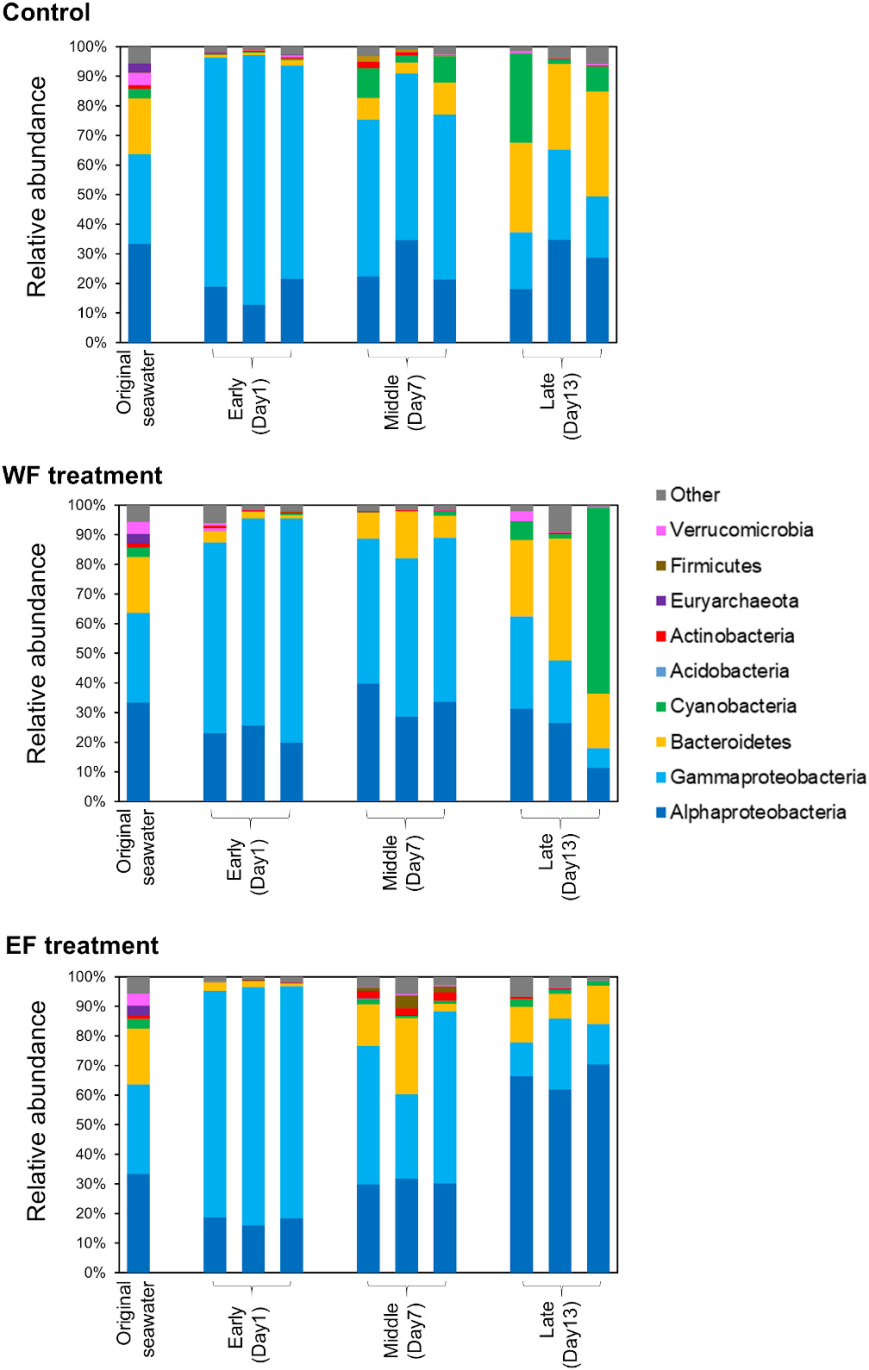
Relative abundance of phylogenetic groups in original seawater and culture samples. Samples from days 1, 7, and 13 are shown. Quality-controlled reads were clustered into OTUs with sequence identity of 99% using VSEARCH (Rognes *et al.*, 2016). These OTUs were classified at the phylum level (class level for *Proteobacteria*) using SINA (Pruesse *et al.*, 2012). In each treatment, data from three replicates are shown.

We then classified all OTUs at the phylum level (class level for *Proteobacteria*) and investigated changes in their relative abundance over the course of the experiment (Fig. 3). In the original seawater sample, *Alphaproteobacteria* (33.4%), *Gammaproteobacteria* (30.2%), and *Bacteroidetes* (18.9%) were the most abundant groups. During the experiment, in the control samples, *Gammaproteobacteria* markedly increased to 77.9% on day 1 and then decreased to 23.5% by day 13. *Bacteroidetes* decreased to 1.3% on day 1 and then increased gradually to a peak at 31.6% by day 13. The WF treatment group showed a similar pattern to that of the control group despite the distinct plot pattern in PCoA (Fig. 2). The EF treatment group showed a different compositional shift on day 1. While *Alphaproteobacteria* in the control and WF treatment groups maintained their abundance at approximately 30% throughout the experiment, *Alphaproteobacteria* in the EF treatment increased their abundance from 17.7% (day 1) to 66.2% (day 13).

**Fig. 4.**
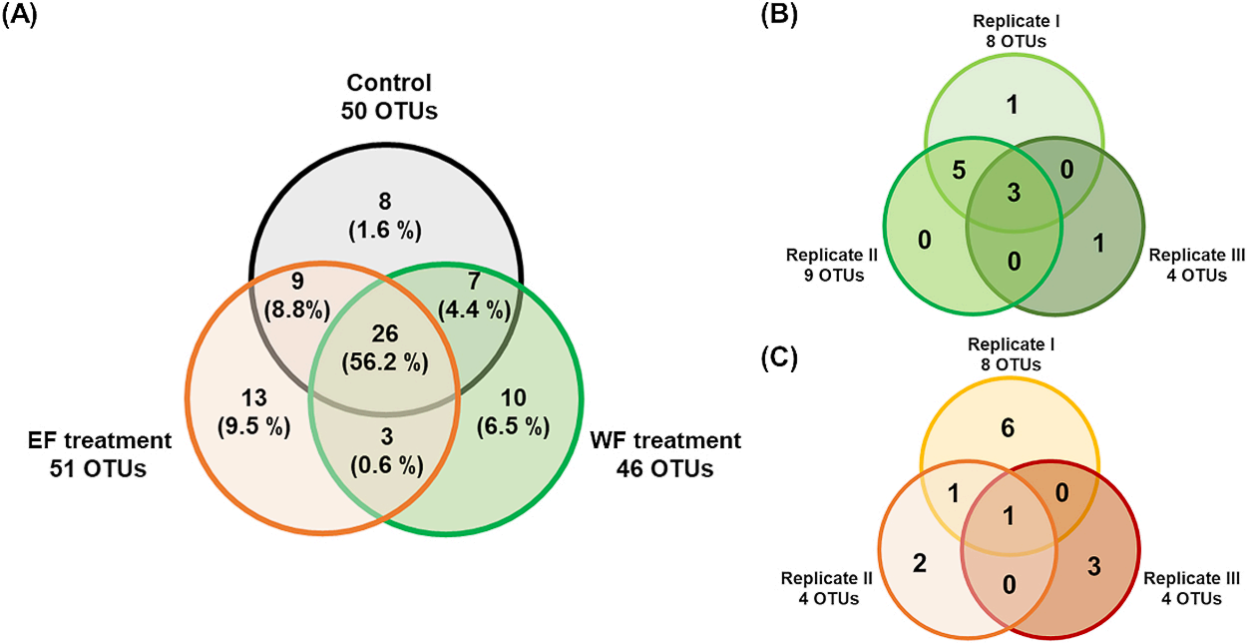
Venn diagram showing the distribution pattern of abundant OTUs. (A) The distribution pattern of all abundant OTUs across treatments. The number shown in the Venn diagram indicates the number of abundant OTUs detected in each treatment. The proportion shown in each area indicates the total of the average abundance during cultivation in each flask (*e.g.* 13 EF-specific OTUs accounted for 9.5% of the relative abundance in each EF flask on average.). (B) The distribution pattern of WF-specific OTUs across replicates. (C) The distribution pattern of EF-specific OTUs across replicates.

We investigated the dynamics of abundant OTUs (relative abundance exceeding 0.4% on average or 2.5% on any sampled day) in each sample over the experimental period. In the original seawater sample, we detected 50 abundant OTUs, and the total relative abundance of these OTUs accounted for more than 60% of this sample (Table S2A). These OTUs included clades of *Alphaproteobacteria* (25.7%), *Gammaproteobacteria* (22.1%), *Bacteroidetes* (12.7%), and *Cyanobacteria* (2.8%). However, 32 OTUs decreased during the cultivation period and were not detected as abundant OTUs in cultures of the samples (Table S2A). In the control, WF treatment, and EF treatment groups, 50, 46, and 51 abundant OTUs were detected, respectively. Among these, 8, 10, and 13 OTUs were specifically abundant in the control, WF treatment, and EF treatment groups, respectively (*i.e.* although these OTUs were detected in the other treatments, their abundance was below the threshold to be considered abundant), and these OTUs were hereafter referred to as treatment-specific OTUs. Twenty-six OTUs were abundant in all treatment groups (hereafter referred to as common OTUs) (Fig. 4) and three OTUs were found to be abundant in both the WF and EF treatment groups, but not in control samples (hereafter referred to as shared OTUs).

The 26 common OTUs accounted for 56.2% of the total relative abundance in each flask on average. Taxonomy assignments using 16S rRNA sequences revealed that 24 out of the 26 common OTUs were heterotrophic bacteria belonging to *Alphaproteobacteria* (7 OTUs), *Gammaproteobacteria* (11 OTUs), *Epsilonproteobacteria* (1 OTU), and *Bacteroidetes* (5 OTUs) (Table S2B). The other 2 common OTUs were assigned to *Cyanobacteria*. When we examined the top hit organisms for the common OTUs using SINA, only 5 OTUs showed species-level similarity (>98.5% identity) with cultured bacteria (Table S2B). Thirteen of the common OTUs were also abundant in the original seawater sample (>0.4%). *Rhodobacterales*, *Oceanospirillales*, and *Flavobacteriales* were dominant at the order level (7, 6, and 5 OTUs, respectively). Changes in the abundance of common OTUs were dynamic in control samples (Fig. S4A) and in WF and EF samples (data not shown). OTU_6 assigned as *Alteromonadales* and OTU_7 assigned as *Rhodobacterales* showed rapid growth from the original seawater sample on day 1 and were the most abundant OTU for up to 6‍ ‍d in all flasks, except for replicate II in the control group.

**Fig. 5.**
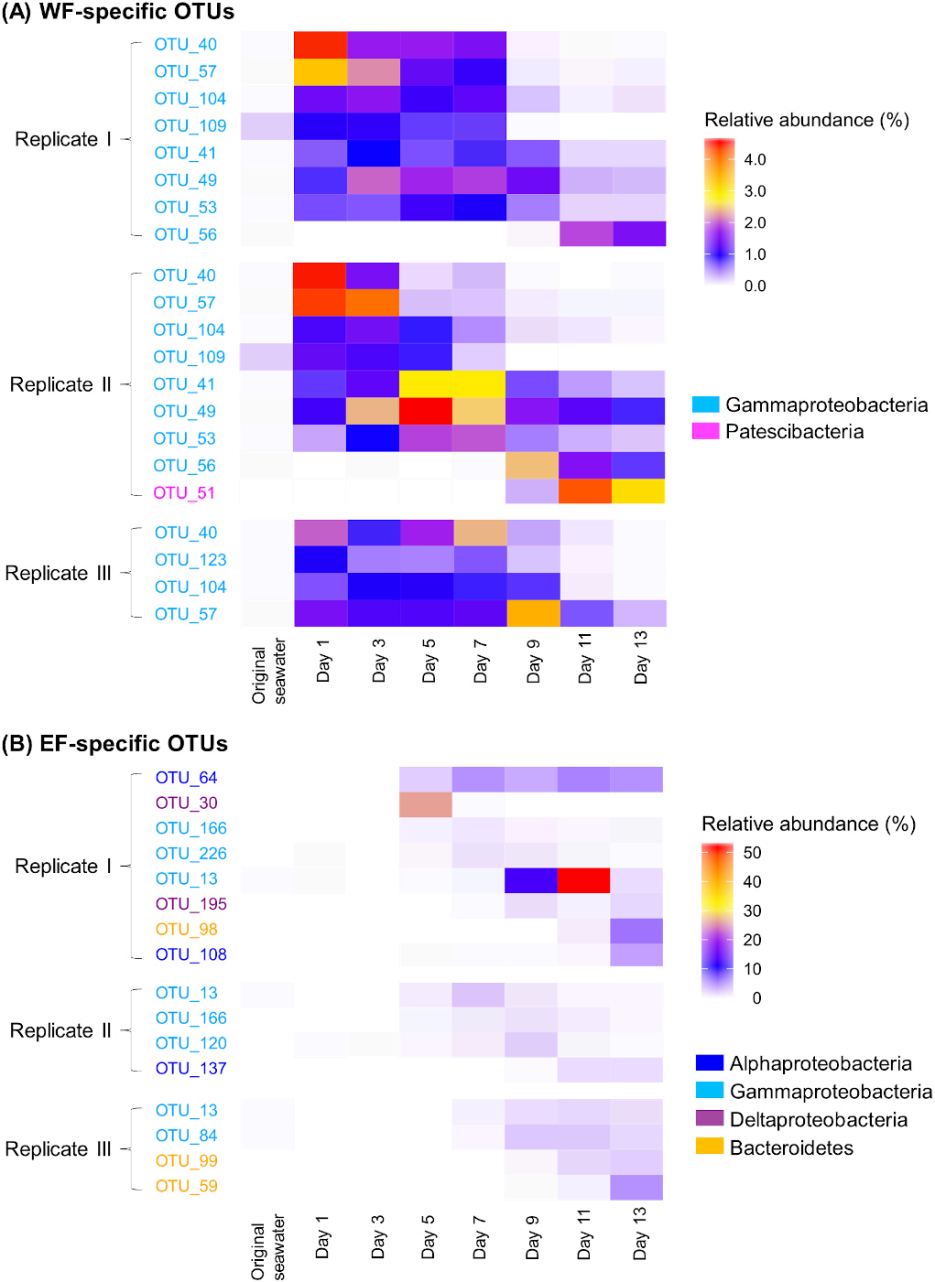
Dynamics of treatment-specific OTUs during culture experiments. (A) WF-specific OTUs. (B) EF-specific OTUs. Samples on day 1 were collected 2‍ ‍d after the collection of the original seawater sample. The color gradient shows the relative abundance. Note that the scale bar in each figure shows the different range of values.

The total relative abundance of the 10 WF-specific OTUs accounted for 6.5% of the total relative abundance in each WF flask on average (Fig. 4A). These OTUs were all heterotrophic bacteria (Table 1A); nine were *Gammaproteobacteria*, of which 6 belonged to *Alteromonadales* and 1 each to *Vibrionales*, *Cellvibrionales*, and *Oceanospirillales*. The remaining WF-specific OTU was classified into the *Patescibacteria* clade. Among these OTUs, only 4 had species-level sequence similarity (>98.5% identity) to cultured bacteria (Table 1A). None of the WF-specific OTUs were abundant in the original seawater sample (<0.2%). When we compared the distribution pattern of WF-specific OTUs across replicates, 80% were abundant in at least two flasks (Fig. 4 and Table 1A). The succession of the WF-specific OTU community was highly dynamic from day 1; six OTUs became the most abundant in each flask by day 13 (Fig. 5A).

The 13 EF-specific OTUs accounted for 9.5% of the relative abundance in each EF flask on average (Fig. 4B). While 90% of WF-specific OTUs belonged to *Gammaproteobacteria*, the 13 EF-specific OTUs were assigned to diverse lineages of heterotrophic bacteria (Table 1B). *Gammaproteobacteria* was the dominant taxon (5 OTUs), and the remaining 8 OTUs were *Alphaproteobacteria* (3 OTUs), *Bacteroidetes* (3 OTUs), and *Deltaproteobacteria* (2 OTUs). Of these, 4 OTUs showed species-level sequence similarity (>98.5% identity) with cultured bacteria (Table 1B). Furthermore, none of these OTUs were abundant in the original seawater sample (<0.2%). The distribution and dynamics patterns of EF-specific OTUs were distinct among flasks. More than 84% of EF-specific OTUs were abundant in only one flask (Fig. 4 and Table S1B). In replicate I, 6 out of 8 EF-specific OTUs were unique to this flask. EF-specific OTUs showed successive dynamics only after the middle phase (after day 5) (Fig. 5B).

Three shared OTUs were assigned as *Flavobacteriales*, *Rhodobacterales*, and *Nitrosococcales* (Table S2C) and accounted for 0.6% of relative abundance in each WF and EF flask on average. These OTUs did not show species-level sequence similarity (>98.5% identity) with cultured bacteria (Table S2C). Although these 3 OTUs were not detected in all flasks, they showed similar dynamics patterns across the WF and EF treatments (Fig. S4B). OTU_55 (*Flavobacteriales*) and OTU_132 (*Nitrosococcales*) were abundant after day 5, while OTU_68 (*Rhodobacterales*) increased after day 7 in both the WF and EF treatment groups.

### Dynamics of abundant OTUs in the natural environment

**Fig. 6.**
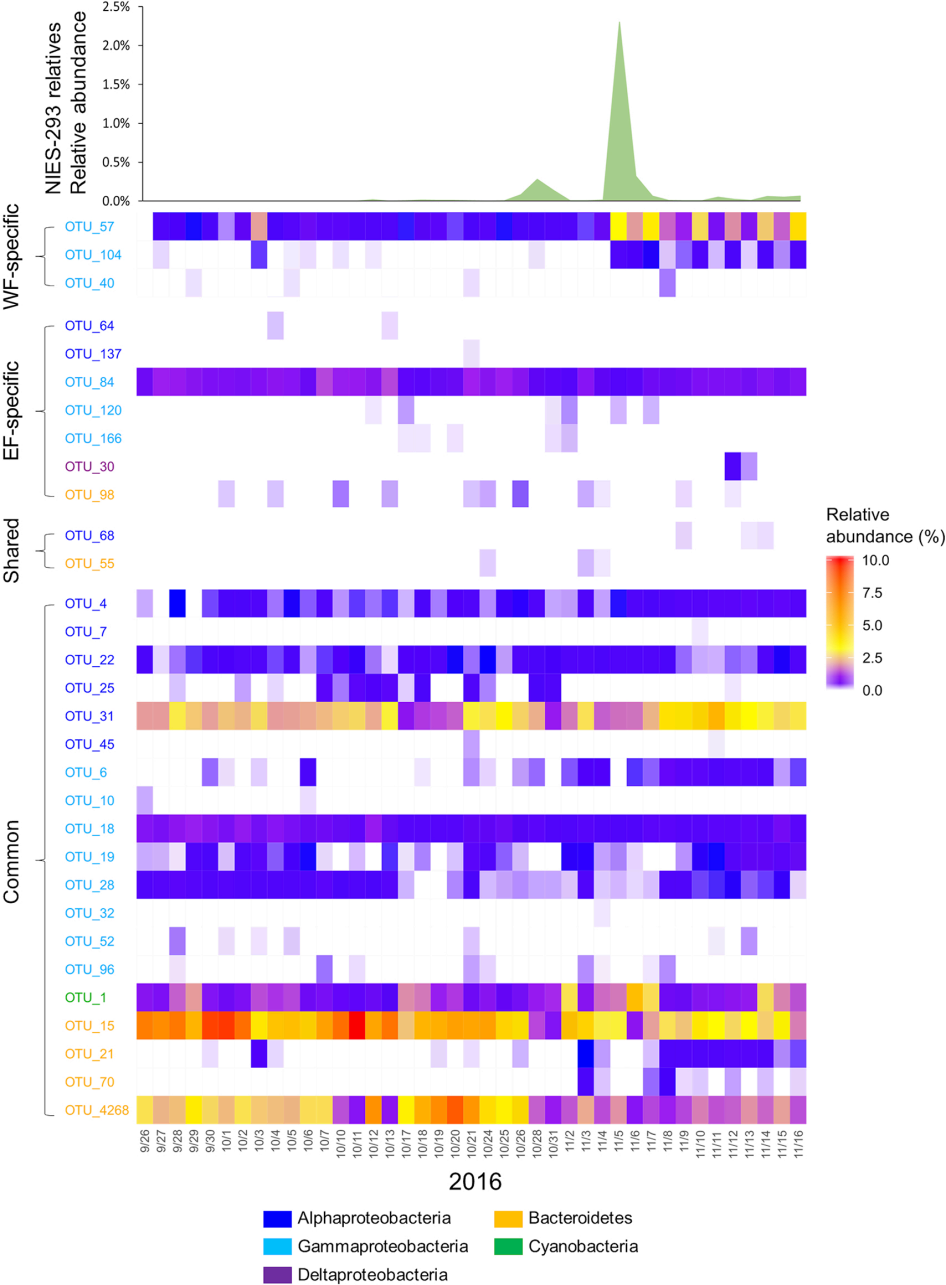
Dynamics of NIES-293 relatives, common OTUs, treatment-specific OTUs, and shared OTUs during a natural phytoplankton bloom. Natural bloom samples were collected in Monterey Bay, USA, between 26th September and 16th November (Nowinski *et al.*, 2019). The 18S rRNA gene sequence of *Heterosigma akashiwo* (NIES-293) was downloaded from GenBank (DQ470658.1). The raw reads of the 16S and 18S rRNA genes in a coastal phytoplankton bloom were obtained from the NCBI Sequence Read Archive (PRJNA533622). Quality-controlled reads were mapped to the sequence of NIES-293 (18S rRNA gene), common OTUs, shared OTUs, and specific OTUs (16S rRNA gene) with 99% identity using VSEARCH. The color gradient shows the relative abundance.

To investigate whether these abundant OTUs emerge in the natural phytoplankton bloom of *H. akashiwo*, we examined the dynamics of close relatives of *H. akashiwo* NIES-293 and the abundant OTUs detected in the present study during a coastal phytoplankton bloom at Monterey Bay, USA between 26 September and 16 November, 2016 (Nowinski *et al.*, 2019). *Dinoflagellata* was the most dominant phytoplankton taxon (>20%), followed by *Protalveolata* and *Ciliophora*. *H. akashiwo* relatives (sharing 99% sequence identity with NIES-293) showed high relative abundance in the samples collected on 5 November (2.3%) (Fig. 6).

During the observed bloom period, 19 common OTUs, 3 WF-specific OTUs, and 7 EF-specific OTUs were detected (Fig. 6). The dynamics of common OTUs showed active succession throughout this period (Fig. 6). In particular, OTU_1 (*Synechococcales*), OTU_15 (*Flavobacteriales*), OTU 31 (*Rhodobacterales*), and OTU 4268 (*Flavobacteriales*) were abundant, with relative abundance peaking at 2.8–10.1%. The bloom-forming period of *H. akashiwo* and the dynamics of common OTUs did not show any co-occurrence patterns. WF- and EF-specific OTUs showed distinct dynamics patterns. Among the detected WF-specific OTUs, OTU_57 (*Alteromonadales*) was abundant on 3 October (2.1%) and from 5–16 November‍ ‍(0.5%~4.1%) (Fig. 6). Although OTU_104 (*Alteromonadales*) did not show high abundance during the observation period (<0.03%), the dynamics of this OTU matched that of OTU_57. The period in which they were abundant in November started on the same day of the *H. akashiwo* bloom. In contrast, EF-specific OTUs did not have a common dynamics pattern (Fig. 6). OTU_84 (*Cellvibrionales*) was the sole dominant OTU throughout the observation period (<1.4%), and none of the other OTUs were abundant (<0.07%). The dynamics of these OTUs did not show co-occurrence with that of *H. akashiwo*. Among 3 of the shared OTUs, OTU_55 (*Flavobacteriales*) and OTU_68 (*Rhodobacterales*) were observed in the natural bloom. OTU_55 increased in abundance before the *H. akashiwo* bloom, while OTU_68 became abundant after the bloom; however, neither were abundant during the bloom (<0.01%) (Fig. 6).

### Phylogenetic classification based on 16S rRNA genes

**Fig. 7.**
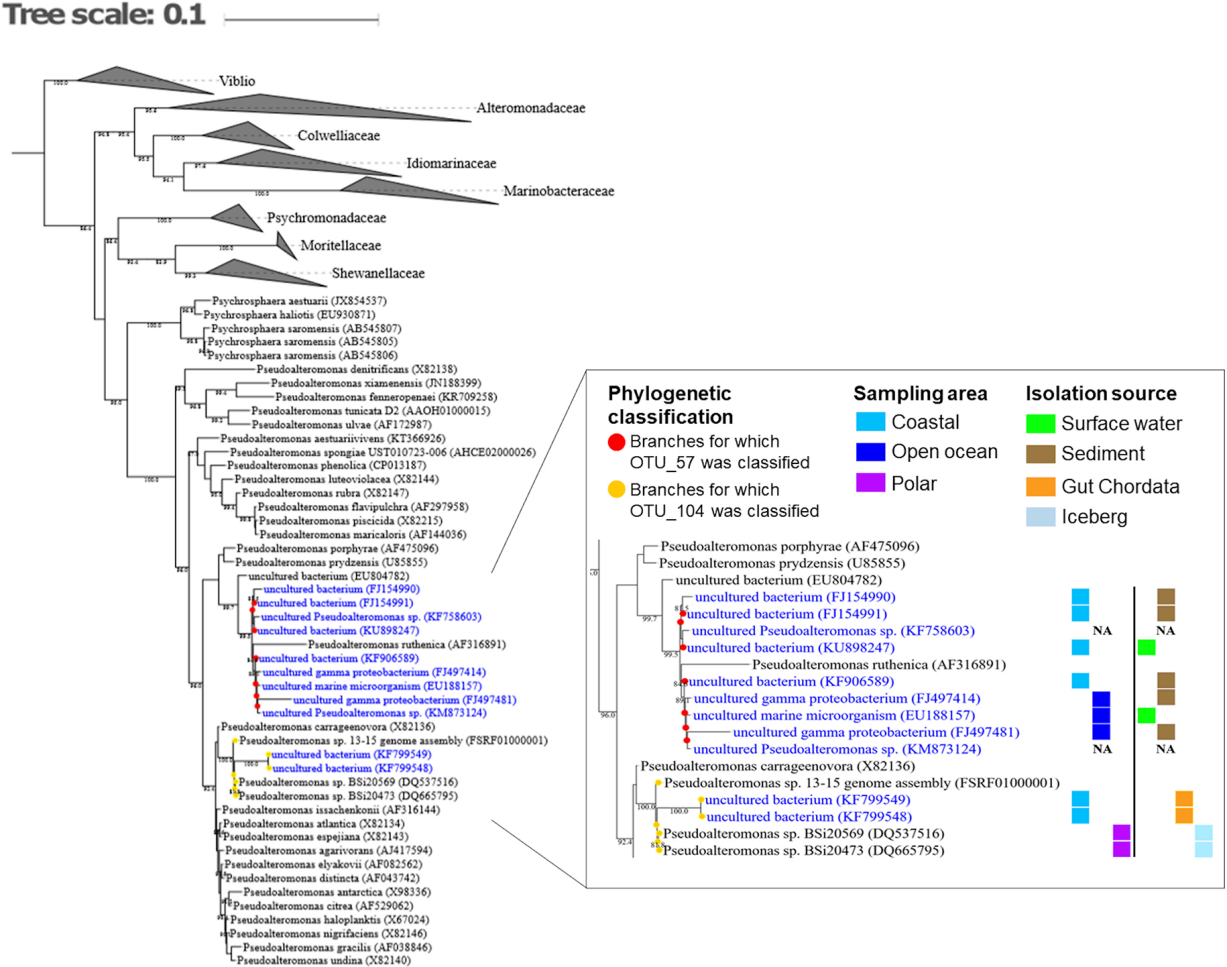
Phylogenetic classification of OTU_57 and OTU_104 among *Alteromonadales* based on the 16S rRNA gene. The reference phylogenetic tree of *Alteromonadales* (*Vibrionales* were included as the outgroup) was constructed using the approximately-maximum likelihood method. The accession numbers of the sequences in the SILVA rRNA database are given in parentheses. Bootstrap values >80% are shown at the roots of all branches. Uncultured bacteria are shown in blue. Colored squares indicate the geographical regions of the origin of the sequences (NA: Not Annotated). Red and yellow circles indicate branches for which OTU_57 and OTU_104 were classified, respectively.

We phylogenetically classified OTU_57 and OTU_104, which showed co-occurrence with *H. akashiwo* in both the culture experiment and environmental samples. OTU_57 showed evenly high similarity with 7 uncultured bacteria, with habitats ranging from the coastal surface water to open ocean sediments (Fig. 7). Taxonomic assignment using SINA revealed that OTU_104 was similar to a cultured *Pseudoaltenomonas* strain (FSRF01000001). The phylogenetic analysis showed that OTU_104 shared 16S rRNA gene sequence similarity evenly with 2 uncultured bacteria and 4 cultured bacteria, which had origins in the polar region and in the gut of chordates.

## Discussion

In the present study, we investigated how the thriving and dead cells of a single species of uninfected phytoplankton influence a coastal prokaryotic community. We prepared WF, representing cellular components released by grazing, and EF, representing exudates of living cells, from the phytoplankton at bloom-modeling cell density in a prokaryotic community. Although the concentration of organic matter in WF and EF may have differed and an incubation bias may have affected the prokaryotic community structure, prokaryotic cell abundance did not significantly differ among treatments. Regarding the prokaryotic inoculation, we defined the 0.2–3.0-μm fraction as the prokaryotic community. Previous studies reported that autotrophic, mixotrophic, and heterotrophic pico-eukaryotic cells were included in this fraction (Christaki *et al.*, 1999; Hartmann *et al.*, 2012; Yang *et al.*, 2018) and we detected a few portions of reads in the 16S rRNA analysis as mitochondrial (it is important to note that the DNA extraction method was optimized for prokaryotic cells). Furthermore, this seawater fraction also included photoautotrophic prokaryote cyanobacteria and photoheterotrophic prokaryotes, such as proteorhodopsin-harboring bacteria. These eukaryotes and phototropic prokaryotes may have affected prokaryotic cells in the three treatments. However, since these organisms exerted similar effects in all three treatments, the preparation of the control treatment enabled us to extract OTUs that specifically responded to changes in other conditions (*i.e.* WF and EF). Therefore, regardless of direct or indirect approaches, we demonstrated that cellular components and exudates from a single species of uninfected phytoplankton differentially influenced a prokaryotic community. It is important to note that since f/2 medium in EF was incubated for 14‍ ‍d as the culture of *H. akashiwo*, the composition of f/2 medium may have differed from that in the control and WF, and it is difficult to deny based only on culture-based experiments that this difference may have affected the prokaryotic community structure. However, we detected the effects of the bloom of *H. akashiwo* on the dynamics of specific OTUs in similar public sequence data obtained from a natural bloom in Monterey Bay (Nowinski *et al.*, 2019). Furthermore, more than half of the abundant OTUs detected in the present study showed no similarities with cultured bacteria, indicating that our experiment allowed us to study interactions among uncultured microbes (Table 1 and Table S2).

The results of both the alpha- and beta-diversity analyses suggested that the prokaryotic community structure changed immediately after the start of the culture in all treatment groups (Fig. 1 and S3A), indicating that a population bottleneck influenced the initial community at the prokaryotic inoculation. However, when we focused on the beta-diversity of samples after the middle phase, while the community structure of samples in the control treatment was not similar, that of samples in the organic matter treatment showed high similarity by treatment (Fig. 2). These patterns indicated that organic matter from *H. akashiwo* shaped the prokaryotic community more selectively than f/2 medium. Discrepancies in plots patterns between the WF and EF treatments implied the different effects of WF and EF on the prokaryotic community. In addition to plot patterns, slight differences in cytograms between the treatments also imply that WF and EF differentially affected the taxonomic composition and/or shape of dominant cells (Fig. S1). Indeed we detected significant changes in the community structure between late samples in the WF and EF treatments in the beta-diversity analysis.

Twenty-six common OTUs were detected in all treatments despite differences in the treatment (Table S2B and Fig. S4A), indicating that these OTUs utilize substrates added to all three groups. Two of the 26 OTUs were autotrophic cyanobacterial OTUs, which would utilize nutrients in f/2 medium. The remaining 24 OTUs were heterotrophic bacteria, which may have survived by consuming organic matter and nutrients produced/released by cyanobacterial photosynthesis and/or bacterial proteorhodopsin-mediated energy generation. The nutrients and scarce organic matter in f/2 medium may also be substrates for these OTUs. *Rhodobacterales*, which was the dominant taxa among the common OTUs (Table S2B), are characterized as “ecological generalists” capable of metabolizing diverse organic carbon compounds (Mou *et al.*, 2008; Newton *et al.*, 2010). Alternately, the 24 heterotrophic OTUs may have scavenged organic matter from the carcasses of other prokaryotes which decreased from original seawater, *e.g.* 3 OTUs belonging to the SAR11 clade were abundant (total of 10.6%) in the original seawater sample, but not in culture samples (Table S2A). Prokaryotic carcasses were previously shown to be a potential supply source for the organic matter pool (Worden *et al.*, 2015).

Ten and thirteen OTUs were specifically abundant in the WF and EF treatments, respectively (Fig. 4). Although the total relative abundance of these OTUs was lower than that of common OTUs (Fig. 4), the presence of specific OTUs indicated that they were capable of utilizing the WF- or EF-derived organic matter added in each treatment. Differences in the dominant taxa and their dynamics patterns support our hypothesis that organic matter from WF and EF had distinct effects on prokaryotes.

In the WF treatment group, *Alteromonadales* was found to be the dominant taxon (6 OTUs) and rapidly increased in all WF flasks (Table 1A and Fig. 5A). This result was consistent with previous findings showing that *Alteromonadales* use a fast-growth strategy to compete for nutrients and organic matter and became dominant in response to the stimulation of organic matter (López-Pérez *et al.*, 2012; López-Pérez and Rodriguez-Valera, 2016) Moreover, *Alteromonadales* have been shown to respond to natural diatom blooms and several mesocosm experiments demonstrated that they grew on organic matter from diatoms and dinoflagellates (Tada *et al.*, 2011; Sarmento *et al.*, 2013; Tada *et al.*, 2017). We herein showed that *Alteromonadales* also took up WF from *Raphidophyceae*. Therefore, we propose that *Alteromonadales* grow rapidly in the early phase of bloom utilizing organic matter from a diverse linage of phytoplankton. Furthermore, the successive dynamics patterns of WF-specific OTUs observed throughout the culture period (Fig. 5A) were consistent with the rapid prokaryotic community variations reported in previous studies on natural phytoplankton blooms (Teeling *et al.*, 2012; Needham and Fuhrman, 2016; Teeling *et al.*, 2016). However, viral lysis also appears to be an essential factor promoting the elution of cellular component-derived organic matter from phytoplankton in the environment (Suttle, 2005). Lysate-derived organic matter often has different chemical properties as a result of metabolic modifications during infection using auxiliary metabolic genes (Rosenwasser *et al.*, 2016). Thus, further studies on the mechanisms by which WF from uninfected and infected cells functions in a prokaryotic community will be beneficial for obtaining a more detailed understanding of phytoplankton-prokaryote interactions.

In contrast, EF-specific OTUs belonged to at least 9 of the order-level bacterial clades (Table 1B). EF-specific OTUs increased only after day 5 in all three flasks (Fig. 5B); however, the succession of these OTUs was detected in this treatment as well as the WF treatment. Previous studies reported that marine bacterial consumption transforms the characteristics of organic matter (Romera-Castillo *et al.*, 2011; Zhao *et al.*, 2019). Thus, these OTUs may have responded to changes in the characteristics of EF organic matter.

Comparisons of the distribution patterns of specific OTUs among flasks revealed that the emergence patterns of specific OTUs were unique to each flask, even when they were treated with the same organic matter (Table 2 and Fig. 4). One explanation for this result is that a population bottleneck made the initial community distinctive. However, this result also implies that the relationship between organic matter and the prokaryotic community is not one to one. This result is consistent with the findings of a previous study, in which coastal bacterial communities were found to form complex relationships with the organic matter released from picocyanobacteria (Zhao *et al.*, 2019).

The presence of shared OTUs (OTU_55, OTU_68, and OTU_132) suggested that several clades of bacteria have the ability to use both WF and EF (Table S2C and Fig. S4B). These results support the complexity of the relationship between organic matter and prokaryotes.

These possibilities for the relationship between organic matter and the prokaryotic community need to be investigated based on chemical characteristics in future studies.

Importantly, using metagenomic approaches, we found that OTU_57 and OTU_104 (*Alteromonadales*), which were classified as WF-specific OTUs, showed highly active dynamics following the bloom of *H. akashiwo* (Fig. 6). This result strongly suggests that these OTUs interact with each other or that *H. akashiwo* affects their natural life cycle in the ocean. Notably, OTU_57 was phylogenetically close to 7 uncultured bacteria that inhabit several coastal areas (Fig. 7). Thus, these uncultured *Alteromonadales* bacteria may have a relationship with phytoplankton in the diverse coastal environment.

We herein demonstrated that cellular components and exudates released from a single phytoplankton strain exerted different effects on a coastal prokaryotic community, suggesting that phytoplanktonic cell death is a factor that‍ ‍alters the prokaryotic community locally associated with phytoplankton. The present results indicated that cellular components were preferentially utilized by *Alteromonadales*, whereas exudates were consumed by a diverse lineage of bacteria. While the WF and EF of phytoplanktonic cells both induce the succession of abundant species, succession patterns were distinctive. Furthermore, we detected 2 of these WF-responding species in a natural bloom of *H. akashiwo*. Therefore, we propose that the release processes of organic matter from phytoplankton cells affect marine carbon cycling via prokaryotic selective consumption. Although the experimental design of the present study requires further modification, such as substrate amounts or culture conditions, to reveal microbial interactions in the environment, the combination of culture experiments and a metagenomic data analysis is a promising method for studying their interactions at high resolution.

## Citation

Takebe, H., Tominaga, K., Fujiwara, K., Yamamoto, K., and Yoshida, T. (2020) Differential Responses of a Coastal Prokaryotic Community to Phytoplanktonic Organic Matter Derived from Cellular Components and Exudates. *Microbes Environ ***35**: ME20033.

https://doi.org/10.1264/jsme2.ME20033

## Supplementary Material

Supplementary Material 1

Supplementary Material 2

Supplementary Material 3

## Figures and Tables

**Table 1.** Taxonomic assignments and emergence patterns across flasks of treatment-specific OTUs. (A) WF-specific OTUs and (B) EF-specific OTUs. The sequences of treatment-specific OTUs were aligned to the SILVA ribosomal RNA gene database (release 132). Emergence patterns of specific OTUs across replicates are indicated using circles. Regarding OTUs that share high identity (>98.5%) with cultured bacteria, the top hit bacterium is indicated, with the corresponding accession number from the SILVA ribosomal RNA gene database.

(A)										
OTU ID	Detection across replicates			Phylum	Class	Order	Family	Genus	Top hit organism (Accession number)	Identity (%)
	I	II	III							
OTU_40	○	○	○	*Proteobacteria*	*Gammaproteobacteria*	*Vibrionales*	*Vibrionaceae*	*Vibrio*		
OTU_41	○	○		*Proteobacteria*	*Gammaproteobacteria*	*Alteromonadales*	*Colwelliaceae*	*Thalassotalea*		
OTU_49	○	○		*Proteobacteria*	*Gammaproteobacteria*	*Alteromonadales*	*Colwelliaceae*	*Thalassotalea*	*Thalassomonas* sp. LMC (KU560494.1)	100
OTU_51		○		*Patescibacteria*	*Gracilibacteria*	*Absconditabacteriales* (SR1)				
OTU_53	○	○		*Proteobacteria*	*Gammaproteobacteria*	*Alteromonadales*	*Colwelliaceae*	*Thalassotalea*	*Thalassotalea euphylliae* (LN849949.1)	100
OTU_56	○	○		*Proteobacteria*	*Gammaproteobacteria*	*Cellvibrionales*	*Porticoccaceae*	*Porticoccus*		
OTU_57	○	○	○	*Proteobacteria*	*Gammaproteobacteria*	*Alteromonadales*	*Pseudoalteromonadaceae*	*Pseudoalteromonas*		
OTU_104	○	○	○	*Proteobacteria*	*Gammaproteobacteria*	*Alteromonadales*	*Pseudoalteromonadaceae*	*Pseudoalteromonas*	*Pseudoalteromonas* sp. 13–15 (FSRF01000001.2772116)	100
OTU_109	○	○		*Proteobacteria*	*Gammaproteobacteria*	*Oceanospirillales*	*Nitrincolaceae*	*Marinobacterium*		
OTU_123			○	*Proteobacteria*	*Gammaproteobacteria*	*Alteromonadales*	*Colwelliaceae*		*Thalassomonas actiniarum* (JYNI01000103.3)	100

(B)										
OTU ID	Detection across replicates			Phylum	Class	Order	Family	Genus	Top hit organism (Accession number)	Identity (%)
	I	II	III							
OTU_13	○	○	○	*Proteobacteria*	*Gammaproteobacteria*	*Cellvibrionales*	*Halieaceae*	*Halioglobus*		
OTU_30	○			*Proteobacteria*	*Deltaproteobacteria*	PB19				
OTU_59			○	*Bacteroidetes*	*Bacteroidia*	*Cytophagales*	*Spirosomaceae*	*Taeseokella*	*Lacihabitans* sp. SM1504 (KU529276.1)	100
OTU_64	○			*Proteobacteria*	*Alphaproteobacteria*	*Rhodobacterales*	*Rhodobacteraceae*		*Phaeobacter* sp. CECT 7735 (CYTW01000009.4203)	100
OTU_84			○	*Proteobacteria*	*Gammaproteobacteria*	*Cellvibrionales*	*Halieaceae*	*Luminiphilus*		
OTU_98	○			*Bacteroidetes*	*Bacteroidia*	*Chitinophagales*	*Saprospiraceae*	uncultured		
OTU_99			○	*Bacteroidetes*	*Bacteroidia*	*Chitinophagales*	*Saprospiraceae*	*Aureispira*	*Aureispira* sp. CCB-QB1 (JHBV01000176.85)	99
OTU_108	○			*Proteobacteria*	*Alphaproteobacteria*	*Rhodobacterales*	*Rhodobacteraceae*			
OTU_120		○		*Proteobacteria*	*Gammaproteobacteria*	*Alteromonadales*	*Colwelliaceae*	*Colwellia*		
OTU_137		○		*Proteobacteria*	*Alphaproteobacteria*	*Rhizobiales*	*Devosiaceae*	*Maritalea*	*Maritalea* sp. LNM-14 (AB758563.1)	100
OTU_166	○	○		*Proteobacteria*	*Gammaproteobacteria*					
OTU_195	○			*Proteobacteria*	*Deltaproteobacteria*	*Bdellovibrionales*	*Bdellovibrionaceae*	*Bdellovibrio*		
OTU_226	○			*Proteobacteria*	*Gammaproteobacteria*	*Oceanospirillales*				
