# Supplementary material for: Differential Responses of a Coastal Prokaryotic Community to Phytoplanktonic Organic Matter Derived from Cellular Components and Exudates
Source: Microbes Environ. 2020 Jun 17;35(3):ME20033. doi: 10.1264/jsme2.ME20033 (PMC7511794; doi:10.1264/jsme2.ME20033)
Supplement: Supplementary file 1 — Supplementary Material 1 [file 35_20033_s1.pdf]

Fig. S1 (Takebe *et al.*)

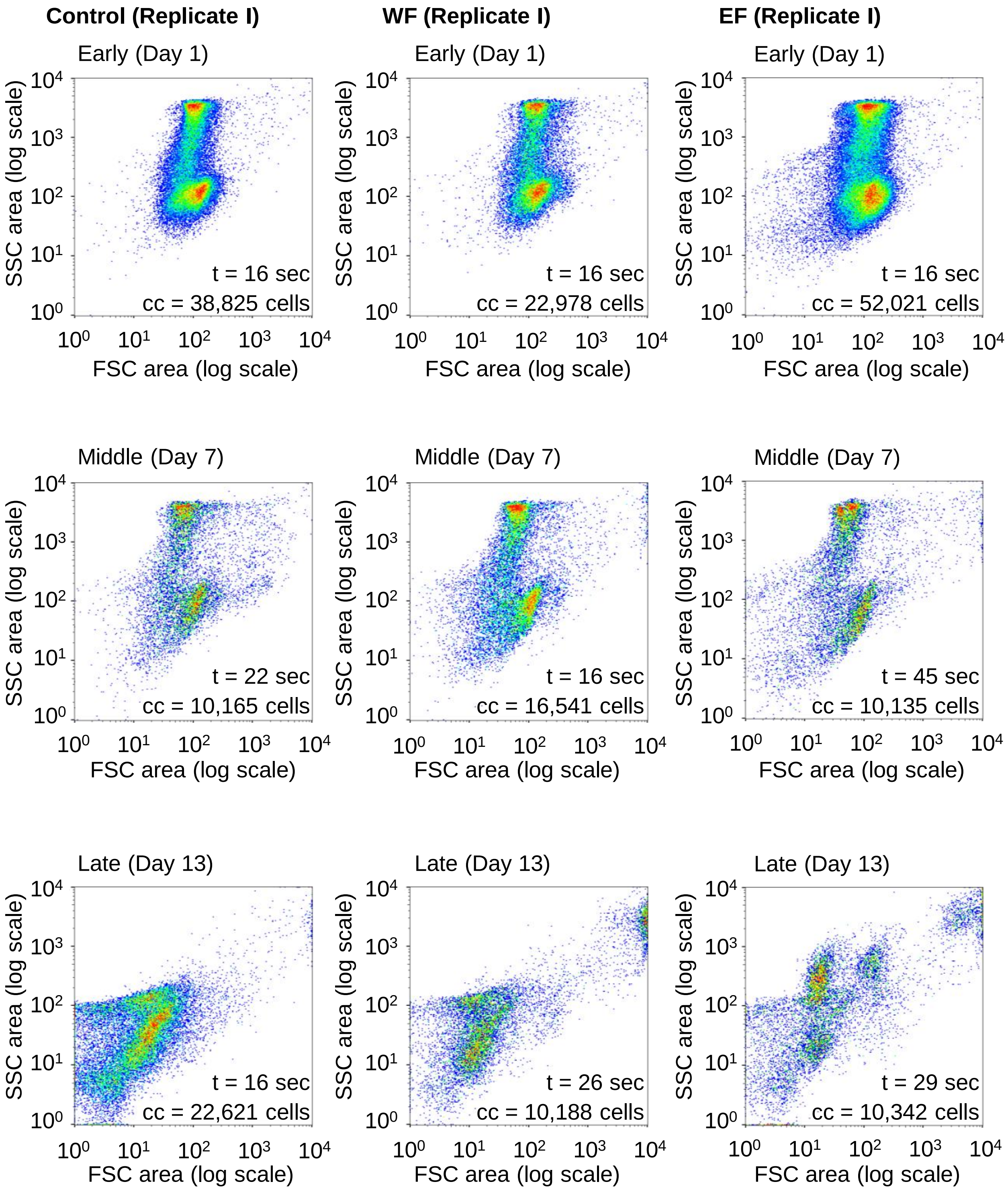

Fig. S1. Density plots of cellular profiles analyzed using flow cytometry. X-axis: forward scatter (FSC), Y-axis: side scatter (SSC), t: measurement time, cc: cell counts. Samples in the early phase (day 1), middle phase (day 7), and late phase (day 13) are shown in the upper, middle, and bottom panels, respectively.

Fig. S2 (Takebe *et al.*)

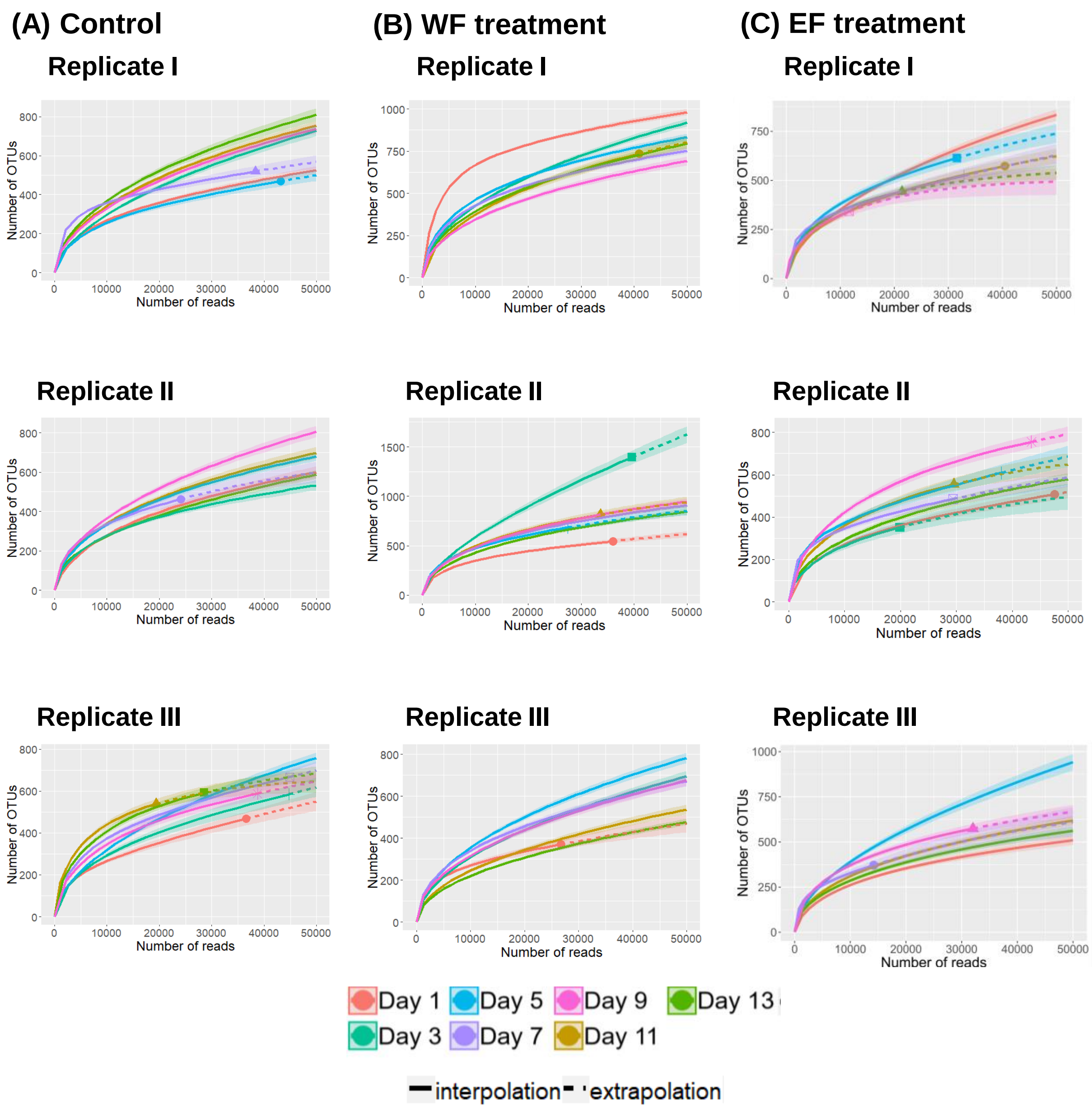

Fig. S2. Rarefaction curves generated from the number of OTUs detected in each sample. **(A)** Control, **(B)** WF treatment, and **(C)** EF treatment. From each sample, 50,000 reads were randomly extracted. Samples with fewer than 50,000 reads were compensated using the "extrapolation" function in "iNEXT". Samples are distinguished by different colors based on the date of collection.

Fig. S3 (Takebe *et al.*)

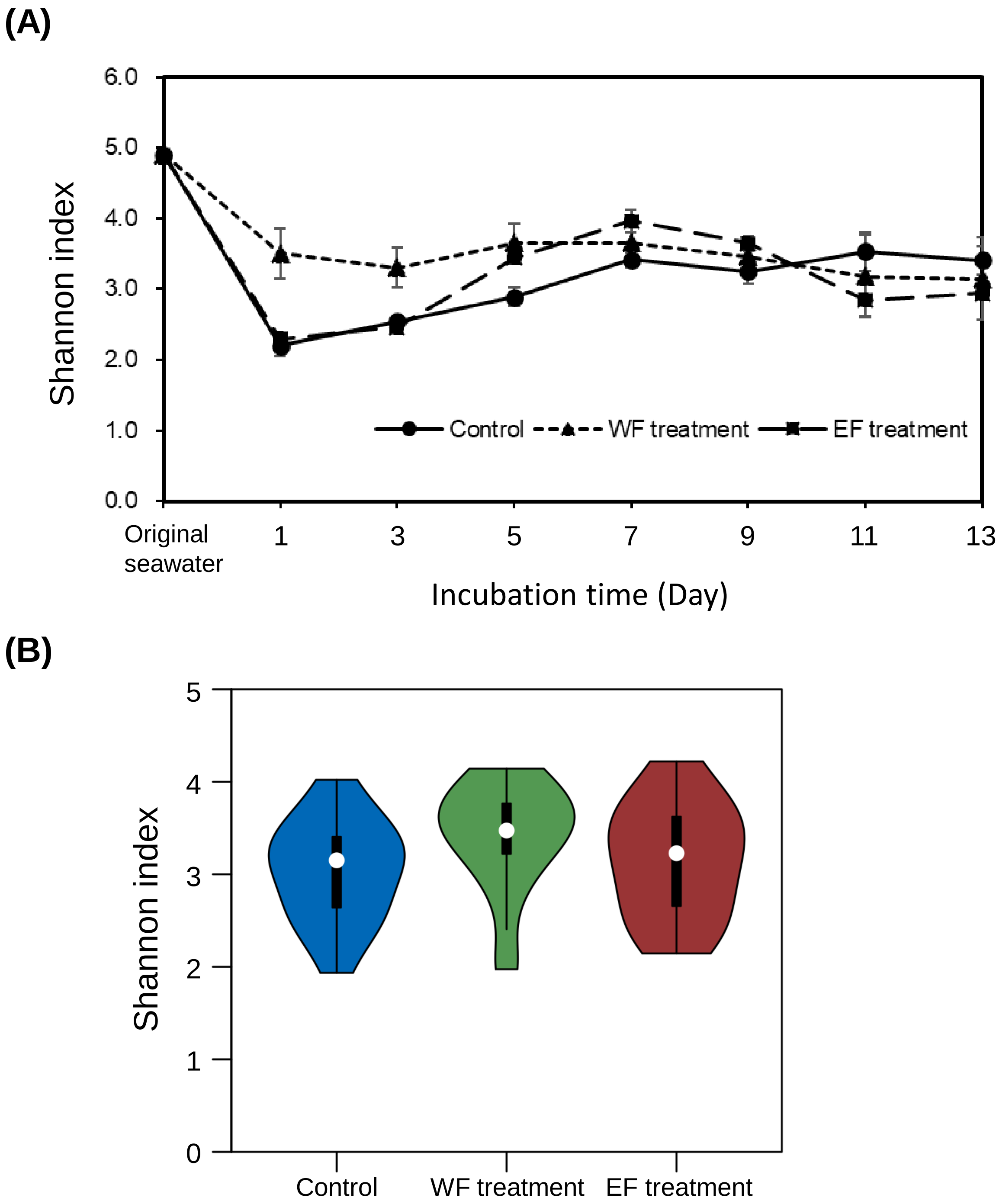

Fig. S3. Shift and comparison of alpha-diversity among three treatments. To normalize the data size among samples, 10,000 reads were first randomly extracted from each sample. Shannon index was calculated using "vegan" package in R. (A) Shift of Shannon index during experiment. Samples on day 1 were collected 2 days after the collection of the original seawater sample. Error bars indicate standard error (For the sample on day 3 in EF treatment, n=1). (B) Violin plots, which were created using "vioplot" package in R, indicating the comparison of Shannon index across treatments. White circles, bounds, and bars represent the median, upper and lower quartiles, and maximum and minimum values, respectively.

Fig. S4 (Takebe *et al.*)

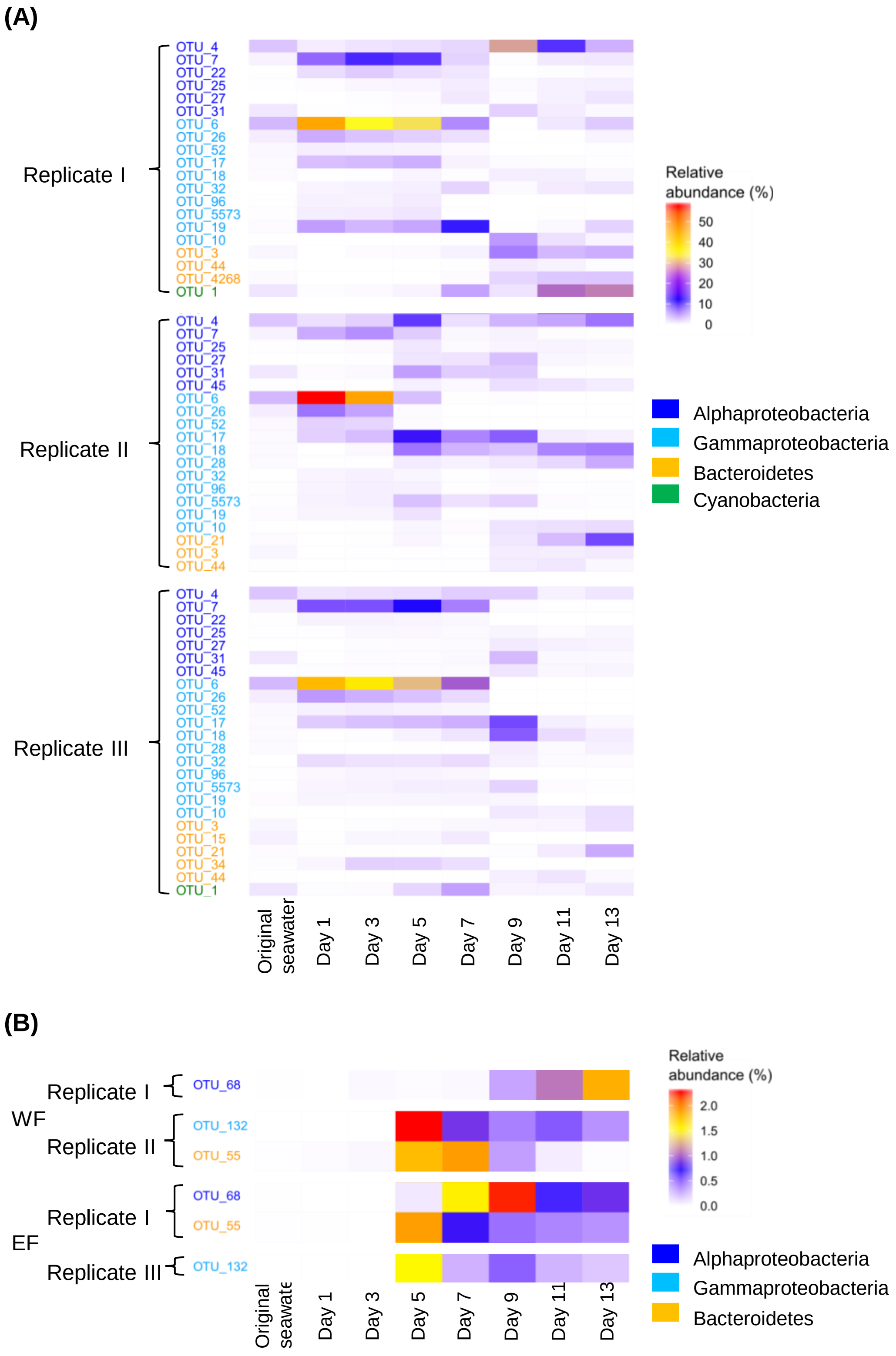

Fig. S4. Dynamics of common OTUs and shared OTUs during the culture experiment. **(A)** Dynamics of common OTUs in control treatment. **(B)** Shared OTUs. Samples from day 1 were collected 2 days after the collection of the original seawater sample. The color gradient shows the relative abundance. Note that the scale bar in each figure shows the different range of values.
